# Zika virus and male reproductive health: essential updates for andrologists and fertility specialists

**DOI:** 10.1186/s12610-026-00305-5

**Published:** 2026-03-11

**Authors:** Ayaz Khan, Michael George, Ian Pearce, Vaibhav Modgil, Theodora Stasinou

**Affiliations:** 1 Department of Urology, Royal Bolton Hospital, Bolton NHS Foundation Trust, Bolton, UK; 2 Department of Urology, Stepping Hill Hospital, Stockport NHS Foundation Trust, Stockport, UK; 3 Manchester Andrology Research Collaborative, Manchester, UK; 4 Department of Urology, Manchester Royal Infirmary, Manchester University NHS Foundation Trust, Manchester, UK

**Keywords:** Zika virus, Male reproductive health, Semen viral persistence, Testicular function, Spermatogenesis, Sexual transmission, Fertility counselling, Assisted reproductive techniques, Virus Zika, Santé reproductive masculine, Persistance séminale virale, Fonction testiculaire, Spermatogenèse, Transmission sexuelle, Conseil en Fertilité, Procréations médicalement assistées

## Abstract

**Background:**

Zika virus (ZIKV), a mosquito-borne flavivirus, remains a concern for reproductive health despite the waning of the 2015–2016 epidemic. Unique among arboviruses, ZIKV can be sexually transmitted, with viral RNA persisting in semen beyond the acute phase, posing potential risks to male fertility and assisted reproduction. This narrative review provides clinicians with a contemporary understanding of ZIKV epidemiology, virology, and its implications for male reproductive care.

**Results:**

ZIKV transmission has stabilised into low-level endemicity in Central and South America, with Europe reporting sporadic travel-associated cases. Persistence in semen is underpinned by infection of immune-privileged testicular tissues, including Sertoli, Leydig, and germ cells, enabling RNA detection for months post-infection even after systemic symptoms resolve. While replication-competent virus is rarely isolated beyond 4–6 weeks, viral RNA has been detected in semen over 180 days in rare cases.

Clinically, ZIKV infection is associated with transient declines in sperm count, motility, and increased DNA fragmentation, likely mediated by inflammation, blood-testis barrier disruption, and impaired testosterone biosynthesis. Human studies suggest recovery of semen quality in most cases, but animal models demonstrate more persistent testicular damage and subfertility, supporting potential long-term reproductive impact.

Current WHO guidance recommends a 3-month deferral from conception for men with confirmed or suspected ZIKV exposure. In assisted reproduction, cryopreserved semen from recently exposed individuals may retain viral RNA, requiring stringent handling and closed-system storage. Routine semen PCR testing is not widely adopted due to sensitivity limitations and inability to distinguish infectivity; thus, risk stratification based on travel and exposure history remains central to decision-making. In clinical practice, this risk stratification typically applies to men undergoing fertility assessment with recent travel to ZIKV-endemic regions, particularly in Central or South America, with compatible symptom history or recent onset infertility.

**Conclusions:**

ZIKV remains a relevant consideration in andrology and fertility practice, particularly in regions with ongoing endemic transmission and in individuals with travel-related exposure. Awareness of its virological properties, reproductive implications, and guidance for pre-conception counselling, laboratory practice, and semen storage is crucial. With appropriate precautions and patient education, most couples can safely proceed with fertility planning following ZIKV exposure.

**Supplementary Information:**

The online version contains supplementary material available at 10.1186/s12610-026-00305-5.

## Résumé

**Contexte:**

Le virus Zika (ZIKV), un flavivirus transmis par les moustiques, reste une source d’inquiétude pour la santé reproductive malgré le déclin de l’épidémie de 2015-2016. Unique parmi les arbovirus, le ZIKV peut être transmis sexuellement, l’ARN viral persistant dans le sperme au-delà de la phase aiguë, présentant de potentiels risques pour la fertilité masculine et la procréation assistée. La présente revue narrative offre aux cliniciens une compréhension contemporaine de l’épidémiologie du ZIKV, de la virologie du ZIKV, ainsi que de ses implications pour les soins reproductifs masculins.

**Résultats:**

La transmission du ZIKV s’est stabilisée en endémicité de faible intensité en Amérique centrale et du Sud, l’Europe rapportant des cas sporadiques liés aux voyages. La persistance dans le sperme est soutenue par une infection des tissus testiculaires immunoprivilégiés, notamment des cellules de Sertoli, de Leydig, et des cellules germinales, permettant la détection d’ARN pendant plusieurs mois après l’infection, même après la résolution des symptômes systémiques. Bien qu’un virus compétent pour la réplication soit rarement isolé au-delà de 4 à 6 semaines, de l’ARN viral a été détecté dans le sperme jusqu’à plus de 180 jours dans de rares cas.

Cliniquement, l’infection par le ZIKV est associée à des baisses transitoires du nombre de spermatozoïdes, de leur motilité, et d’une augmentation de la fragmentation de l’ADN, probablement médiées par l’inflammation, par la rupture de la barrière sanguin-testicule, et par une biosynthèse altérée de la testostérone. Des études humaines suggèrent une récupération de la qualité du sperme dans la plupart des cas, mais les modèles animaux montrent des lésions testiculaires et une hypofertilité plus persistantes, ce qui soutient un impact reproductif potentiel à long terme.

Les recommandations actuelles de l’OMS sont de reporter de 3 mois la conception pour les hommes ayant une exposition confirmée ou suspectée au ZIKV. En cas d’assistance médicalisée à une procréation, le sperme cryopréservé provenant d’hommes récemment exposés peut retenir de l’ARN viral, nécessitant une manipulation rigoureuse et un stockage du sperme en système fermé. Le test PCR de routine du sperme n’est pas largement adopté en raison de limitations de sensibilité et de l’incapacité à distinguer l’infectiosité ; ainsi, la stratification du risque basée sur l’historique des voyages et des expositions reste centrale dans la prise de décision. En pratique clinique, cette stratification du risque s’applique généralement aux hommes ayant eu une évaluation de leur fertilité en raison de voyages récents dans des régions endémiques du ZIKV, en particulier en Amérique centrale ou du Sud, et qui présentent des antécédents de symptômes compatibles ou une infertilité à début récent.

**Conclusions:**

Le ZIKV reste une considération pertinente en andrologie et en médecine de la fertilité, en particulier dans les régions à transmission endémique continue et chez les individus à risque d’exposition liée aux voyages. Une sensibilisation est cruciale à ses propriétés virologiques, aux implications reproductives, aux conseils lors de consultations pré conceptionnelles, à la pratique en laboratoire, et au stockage du sperme. Avec des précautions appropriées et une éducation des patients, la plupart des couples peuvent poursuivre en toute sécurité la planification d’une grossesse après une exposition au ZIKV.

## Introduction

Zika virus (ZIKV), an arthropod-borne flavivirus first identified in Uganda in 1947 [1], gained particular attention as a global health concern following the outbreak in Central and South America in 2015–2016 [2]. Case numbers have since fallen, but ZIKV remains an ongoing concern with it having now settled into a pattern of low-level circulation in parts of Central America, the Caribbean and Southeast Asia, with sporadic cases continuing to be detected in non-endemic regions such as Europe and the United Kingdom [3]. At the peak of the 2015–2016 outbreak, more than 800,000 suspected and confirmed ZIKV cases were reported across the Americas, with Brazil accounting for the majority of infections; since then, surveillance data demonstrate substantially lower but persistent annual case numbers consistent with endemic circulation [2, 4]. This shift from a high-profile epidemic to quieter, background endemicity poses challenges to reproductive care, particularly for couples with active fertility goals or treatment.

ZIKV is distinguished from most other arboviruses by its capacity for sexual transmission, as it’s RNA may persist in the semen of affected individuals for weeks to months beyond the acute phase, which is often longer than in blood or urine. This characteristic carries two key male sexual health implications - onward transmission to a partner and risk of male sub- or in-fertility [5]. Early in the American outbreak, ZIKV was commonly discussed alongside other widely recognised congenital pathogens (such as those included in the TORCH group: toxoplasmosis, rubella, cytomegalovirus, herpes simplex virus, and other vertically transmitted infections), acknowledging its associated reproductive risk [6]. Since then, human and animal studies have shown that ZIKV can infect multiple testicular cell types, including germ cells, Sertoli cells and Leydig cells, with downstream effects on spermatogenesis, testosterone biosynthesis and testicular architecture [7]. The precise clinical implication on long-term fertility is yet to be clarified but current evidence justifies maintaining ZIKV in the differential in men at clinical risk presenting with subfertility, or when counselling about assisted reproductive techniques.

This narrative review aims to provide a contemporary update on ZIKV epidemiology and biological basis relevant to reproductive health, with discussion of its implications on andrology and reproductive medicine practices. In doing so, we aim to raise awareness of ZIKV amongst clinicians and patients, with practical recommendations for each stage/setting of fertility care.

## Methods

**Fig. 1:**
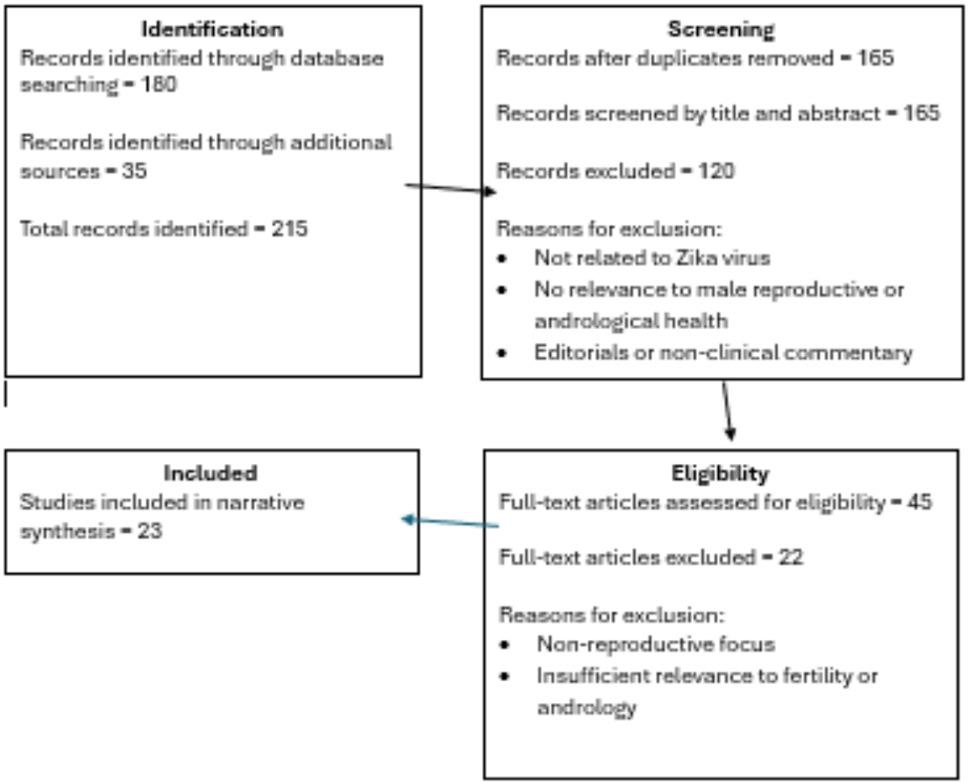
Literature identification and selection for narrative review. Flow diagram summarising the identification, screening, eligibility assessment and inclusion of studies relevant to Zika virus and male reproductive health

This narrative review adopted a structured literature search to identify relevant publications concerning ZIKV and its implications for male reproductive and andrological health (Fig. 1). Searches were primarily undertaken through PubMed which served as the main biomedical database, complemented by targeted searches of international public health agency websites including those of the World Health Organization (WHO), Pan American Health Organization (PAHO), European Centre for Disease Prevention and Control (ECDC), UK Health Security Agency (UKHSA) and Centres for Disease Control and Prevention (CDC). The review draws upon publications spanning a ten-year period, from 2015 to 2025.

Core search terms central to the review’s scope such as “Zika virus”, “male reproductive health”, “fertility”, “andrology” and “assisted reproduction”, were combined with more specific biological terms including “semen”, “testosterone”, “spermatogenesis” and “sexual transmission”. Boolean operators were applied to refine the search and incorporate additional modifiers such as “epidemiology” and “WHO guidance”, to capture both clinical and public health perspectives comprehensively.

Reference lists of selected papers were screened to identify additional relevant material. Both primary research studies and review articles were included alongside epidemiological summaries and guidance documents from recognised health agencies. While no formal inclusion or exclusion criteria were applied, only English-language, accessible, and thematically relevant sources were retained. Publications outside the review’s clinical or reproductive focus were excluded.

Evidence gathered through this process was synthesised narratively and organised under thematic headings aligned with the structure of the review, namely epidemiology, viral persistence in semen, effects on testicular function, and implications for fertility practice. The full electronic search strategy, including exact Boolean search strings and a summary of public health agency sources consulted, is provided in Supplementary Appendix 1.

### Epidemiological update

A decade on from its American outbreak peak in 2015–2016, ZIKV activity has reportedly stabilised into a familiar arboviral pattern - low-level, seasonal transmission in endemic zones with occasional clusters. Between 2019 and 2023, annual reported ZIKV case numbers across the Americas ranged from approximately 30,000 to over 55,000 cases per year, with marked inter-annual and seasonal variability reflecting vector density and climatic conditions [4]. The Americas, particularly Central and South America, continue to account for much of the disease burden; surveillance data from Brazil and neighbouring countries show recurrent upticks aligned with Aedes aegypti breeding and rainy seasons [8] (Table 1). Southeast Asia remains another steady source of cases, with Thailand frequently implicated in travel-associated infections [4].

**Table 1:** Recent Global and Regional Epidemiology of Zika Virus Infection (2019–2023) [4]. Reported Zika virus (ZIKV) cases across selected regions and countries between 2019 and 2023, illustrating current patterns of low-level endemic transmission and sporadic outbreaks. Case numbers are derived from national surveillance data and international public health agencies and may underestimate true incidence due to asymptomatic infection and variable reporting practices

Region/Country	Year	Reported Zika Cases	Notes
Americas (total)	2023	55,813	11% lab-confirmed; ~97% from Brazil.
Brazil	2023	~ 54,000	Major contributor to regional burden; seasonal pattern.
Bolivia	2023	881	Increase from 190 cases in 2022.
Thailand	2023	758	Ongoing endemic transmission; 33 pregnant women affected.
Singapore	2023	30	Small urban cluster; contained by vector control.
India	2023	Sporadic clusters	Recurrent outbreaks in southern and northern states.
Maldives	2023	2 (traveller-linked)	Imported infections confirm local transmission potential.
Mali	2023	22	First locally acquired cases confirmed.
Nigeria	2022	6	RT-PCR confirmed in pregnant women.
Europe	2019	3	Single local cluster in France; otherwise imported cases.

The WHO have illustrated ZIKV trends, suggesting that proliferation and propagation may continue wherever competent vectors are present [1]; in doing so, the WHO underscore the potential reproductive health risks in endemic areas. The CDC similarly advise clinicians to consider countries with current or past ZIKV transmission cases as potential exposure risk, a measured stance in light of under-reporting and the possibility that outbreaks are recognised retrospectively. The CDC’s country and territory list, updated in 2025, remains a useful source for pre-travel counselling and pre-conception planning [9].

In Europe, ZIKV remains primarily a travel-associated diagnosis. The ECDC documents sporadic cases in returning travellers each year without evidence of onward local transmission [10]. In England, a handful of Zika diagnoses were established in the first six months of 2025, with most linked to recent travel to Southeast Asia, mirroring patterns seen in previous years [11]. For clinicians in non-endemic settings, risk stratification should focus on recent travel to regions with ongoing or recent ZIKV transmission (particularly Central and South America, the Caribbean, Southeast Asia, and parts of South Asia and sub-Saharan Africa) alongside symptom history and timing of fertility assessment [4, 9].

### Pathophysiological determinants of viral persistence in testicular tissue

ZIKV’s persistence in semen reflects a convergence of immune privilege, target-cell susceptibility and viral immune-evasion strategies. The testes maintain a locally immunosuppressive setting to safeguard germ cells, with paracrine signalling and specialised macrophage populations that restrain inflammation. This arrangement, beneficial for fertility, can be co-opted by pathogens and ZIKV capitalises on this [12]. Much of the mechanistic understanding underpinning these processes is derived from experimental and animal model studies, with more limited confirmatory data available from human tissue and clinical cohorts.

Sertoli cells, central to germ-cell support and blood-testis barrier (BTB) maintenance, appear permissive to ZIKV infection. Infected Sertoli cells can harbour replication, secrete inflammatory mediators and compromise tight junctions within BTB, exposing germ cells to both infection and bystander injury [6, 13]. Leydig cells are also implicated; experimental studies show that infection lowers testosterone production with evidence suggesting that the viral NS2A protein disrupts normal steroid hormone synthesis [7]. Once the BTB is breached, intratubular spread is facilitated and clearance becomes more difficult.

At the molecular level ZIKV non-structural proteins antagonise type I interferon signalling and mitochondrial antiviral pathways, blunting the local antiviral state [12, 13]. In Sertoli cells, these effects may be amplified by baseline immunoregulatory biases of the testis [6]. Together, immune privilege, long-lived infected cell types, a weakened interferon response and structural barrier disruption explain why RNA can be detected for months after illness, even when infectious virus is no longer recoverable [7, 12].

### Temporal and virological dynamics of ZIKV in seminal fluid

ZIKV’s activity within the male reproductive tract is distinctive. The virus can localise to the testes (Sertoli cells, Leydig cells and germ cells), epididymis, seminal vesicles and prostate, with its RNA detectable in both cellular fractions and seminal plasma [6, 12]. Interestingly, prolonged persistence of viral ZIKV RNA has been observed within the reproductive tract when compared with other body fluids and with other flaviviruses [12]. This tissue tropism underpins ZIKV’s particular preponderance for sexual transmission. Evidence for tissue localisation and cellular mechanisms of persistence is derived predominantly from experimental and animal model studies.

The duration and variability of seminal ZIKV RNA persistence have been described across human cohort studies and systematic reviews. A study by Pley et al. estimated a median persistence of approximately two months (57 days), with the 95th percentile reaching approximately 3–4 months and rare outliers extending beyond this timeframe [5]. In a large multicentre cohort study, RNA remained detectable in some men for up to 6 months although most cases resolved within three [14]. These findings are comparable with earlier prospective studies such as that amongst symptomatic men in Puerto Rico that demonstrated mean semen clearance within 54 days [15], whilst other research has less commonly reported detection beyond 80 days in cases [16]. Published case reports within the literature have also described intermittent positively identifiable RNA beyond 180 days in rare cases [17], suggesting that there may be instances in which a single negative test result may be at risk of misleading clinical decision-making.

Two phases are often inferred in ZIKV propagation. In the initial 30–45 days after symptom onset, rates of RNA identification are high and copy numbers can be substantial, aligning with the period of greatest infectivity. Thereafter, a more prolonged period of low-level, intermittent detection may follow in a smaller subset of men, which may reflect infection of long-lived or immune-shielded testicular cells coupled with limited local immune surveillance [6, 12].

Crucially, findings from human studies demonstrate that RNA detection does not necessarily equate to infectious virus. While RT-PCR remains sensitive for months, replication-competent ZIKV has rarely been isolated beyond 4–6 weeks post-infection in culture-based assays [15, 16], and the multicentre cohort did not recover infectious virus from months-positive specimens [14]. This divergence between RNA and infectivity underlies the current, relatively conservative, timing recommendations for conception and assisted reproduction.

### Effects on sperm and testicular health

Human data suggest that ZIKV can transiently disturb semen quality. In some cohorts, prolonged RNA shedding has coincided with reduced sperm concentration and motility, and with evidence of increased DNA fragmentation, though sample sizes have been modest and follow-up limited [16]. These observations align with plausible hormonal and cellular mechanisms identified primarily in experimental and animal studies, including ZIKV NS2A-mediated disruption of CYP17A1 translation in Leydig cells, impairing testosterone biosynthesis, a pathway that could compromise germ-cell maturation and accessory-gland function and in turn depress semen parameters [7].

Inflammation offers a second mechanism by which ZIKV may adversely affect fertility prospects. ZIKV infection of Sertoli cells and germ cells can provoke a local cytokine response, disturb the seminiferous epithelium and degrade the BTB [18]. Even in the absence of overt orchitis, subclinical damage (defined as inflammatory or structural disruption not associated with clinical symptoms) could explain transient drops in count or motility after apparent clinical recovery [6]. Whether these changes have lasting consequences for fertility in humans remains uncertain; most men appear to recover within months [16], but carefully designed, longer-term studies are desirable. At present, available human data are insufficient to draw firm conclusions regarding long-term fertility outcomes or risks such as recurrent pregnancy loss, beyond supporting cautious follow-up and observation.

Findings in published animal studies appear more conclusive. In mouse subjects, ZIKV replicates within Sertoli cells, disrupts the BTB, reduces testicular volume and testosterone levels, and leads to sustained sub-/in-fertility that persist long after systemic clearance [13]. Non-human primate data support aspects of this clinical picture, with testicular infection and inflammation accompanied by prolonged RNA detection in semen [19]. Although not directly translatable to human outcomes, these models support the biological plausibility of potential reproductive effects, while highlighting the current uncertainty regarding their clinical significance in humans.

### Clinical considerations before sperm retrieval and cryopreservation

From a mechanistic perspective, ZIKV demonstrates tropism for the male reproductive tract through infection of Sertoli, Leydig and germ cells within an immune-privileged environment, with disruption of the blood-testis barrier facilitating viral persistence independent of systemic viraemia [19, 20]. In the context of assisted reproduction, this raises theoretical concerns regarding cryopreservation, as freezing does not reliably inactivate all viral pathogens and may allow prolonged preservation of viral RNA or potentially infectious material. Although direct evidence of ZIKV transmission via cryopreserved semen remains limited, these mechanistic considerations underpin current precautionary laboratory practices, including closed-system cryostorage, sample segregation and enhanced informed consent following recent or suspected exposure [19, 20].

Routine semen screening for ZIKV is not widely adopted and presents practical hurdles. RT-PCR can detect RNA in semen, but inhibitors in seminal plasma, variable viral loads and sampling variability limit sensitivity. Conversely a positive result, as discussed, does not establish the presence of infectious virus [5, 21]. In day-to-day practice, exposure history and timing from infection or travel may yield more value in the decision-making process than a one-off semen PCR.

Timing recommendations are intentionally simple to aid their translation and application in clinical practice. Current WHO guidance advises that men with possible ZIKV exposure delay attempts at conception, natural or assisted, for three months, and women for two months after exposure or illness. These recommendations are precautionary, population-level measures rather than individualised risk estimates [22]. These intervals reflect the observed tail-off of RNA persistence, as well as the narrower window in which infectious viruses have been recovered [14, 15]. These guidelines also balance risk-reduction with practicality for couples.

ZIKV biology also carries significance for sperm storage. While direct evidence of infectious virus persisting through cryostorage in humans is limited, ZIKV can survive freeze-thaw, and viral RNA, and potentially infectious material during early convalescence, may persist through the cryostorage period [12]. Best practice therefore recommends closed-system cryostorage, strict specimen handling and segregation protocols. Unlike other viruses of significance such as HIV, where validated sperm-washing protocols have transformed risk management, no processing method has been proven to reliably and consistently eradicate ZIKV from semen specimens.

In urgent fertility preservation (such as prior to gonadotoxic therapy), it is advisable to document the patient’s exposure history carefully, highlighting residual uncertainties clearly and obtaining informed consent that reflects the balance of risks and time constraints. Where feasible, it may be helpful to consider deferring cryopreservation until outside the early convalescent window.

### Couple counselling and precautionary measures

For couples planning pregnancy after potential exposure, deferral of conception represents the cornerstone in risk management and optimisation of fertility potential, with a recommended three months for men and two months for women from time of symptom onset or latest conceivable exposure, in line with WHO guidance [22]. During this deferral period, couples are advised on safe sexual practices including the consistent use of condoms or abstinence to minimise the risk of sexual transmission. Potential ZIKV exposure may occur through travel to or residence in endemic regions where Aedes mosquitoes are present, unprotected sexual contact with an infected or recently exposed partner, transfusion of contaminated blood products, or occupational contact with infectious materials [1].

Assisted reproductive techniques introduce further practical considerations. Theoretically, the sperm of pre-exposure semen offers the safest option with greatest fertility potential; however, this requires significant forward planning and presents logistical challenges for those embarking on foreign travel. If this is either not available or not in line with patient preference, a negative semen PCR may offer some reassurance but is unable to exclude low-level viral load and is furthermore not able to accurately distinguish between cases that are infectious or not [5, 21]. In time-sensitive scenarios, such as oncofertility, patients should be counselled to understand that deferred conception is considered best practice in view of these limitations associated with testing and sperm washing evidence-base.

It is also important to educate patients on the potential maternal and foetal implications of ZIKV infection, which should form a cornerstone of the counselling process. Sexual transmission from an affected male to their pregnant partner risks congenital Zika syndrome, characterised by microcephaly, limb contractures, and ocular or auditory abnormalities, with long-term consequences including neurodevelopmental delay and sensory impairment [1] Neurological complications such as Guillain-Barré syndrome have been reported in adults with ZIKV infection and are distinct from congenital manifestations [1]. Most women with ZKV infection are asymptomatic; when present, symptoms are mild and short-lived, including rash, low-grade fever, conjunctivitis, arthralgia, malaise, and headache. Infection during pregnancy may lead to complications such as fetal loss, stillbirth, or preterm birth [1]. For these reasons, individuals are once again encouraged to use condoms or abstain for the pregnancy whilst within the deferral or RNA-positive window [9]. Couples who observe the recommended deferral period as advised can usually proceed safely thereafter.

### Clinical relevance of ZIKV in 2026

As endemic circulation continues in Latin America and Southeast Asia, with imported cases recorded annually in Europe and the UK, ZIKV represents an ongoing concern amongst andrologists and fertility specialists [9, 14]. Whilst the trajectory of ZIKV numbers is challenging to predict, factors such as climate change, urbanisation and the spread of Aedes vectors increasingly provide conditions for its transmission. Furthermore, it has been suggested that an enzootic cycle in non-human primates may also act as a dormant reservoir with the potential to re-seed future human outbreaks under conditions [12]. Whilst no ZIKV variant has progressed to dominance over its counterparts, viral evolution theorises the potential for new strains in future that may warrant ongoing surveillance.

Within reproductive care a proactive, rather than reactive, approach is preferrable. In order to promote high quality, patient-centred care, a considered multi-disciplinary approach is required to identify potential exposures and educate patients on appropriate precautions. Patients may benefit from a patient information resource that they can turn back to at a later date. It is therefore essential that patients and clinicians alike maintain a high index of suspicion for ZIKV and are aware of its potential implications.

### Future directions

Despite advances in virology and ZIKV care, there remain areas deserving of future attention.

It is essential that the role of ZIKV testing, encompassing PCR and culture, is established and procedural techniques optimised for clinical practice. At present, a positive seminal PCR does not confer an infectious state, whilst culture is slow, insensitive and rarely positive late after infection [23]. There may be a role for novel surrogates of viral activity, for example molecular/metabolic signatures, antigen detection panels, or validated thresholds that predict transmission risk more accurately than a binary PCR read-out [5, 13, 15].

Similarly, there may be a role to consider the most appropriate biological matrix for testing. It may be hypothesised that assessment of seminal data offers greatest yield, given its proximity to the reproductive tract and fertility prospects, however some clinical laboratories are not validated for semen processing in view of its challenging standardisation and external quality-assurance [24]. Developing robust, semen-adapted assays may enable more individualised risk assessment and individualised advice, in turn reducing the reliance on blanket waiting periods [21].

Rodent and primate studies have demonstrated convincing testicular injury and reduced fertility even months after infection [13, 19]. To date human cohorts, however, are challenged by small sample sizes and limited follow-up. Large, prospective studies that track semen parameters, testosterone levels and reproductive outcomes, whether that be via natural conception or ART, would enable improved understanding of the natural history of the condition and once again support tailored care.

### Limitations of the study

This review has several limitations that should be acknowledged. First, as a narrative review, the literature selection and synthesis were qualitative rather than systematic, and while a structured search strategy was employed across major biomedical databases and international public health agencies, formal risk-of-bias assessment and quantitative meta-analysis were not undertaken. Second, much of the mechanistic understanding of ZIKV persistence within the male reproductive tract, including effects on spermatogenesis, steroidogenesis and testicular architecture, is derived from animal models and in vitro studies, which may not fully translate to human reproductive outcomes. Human data remain limited, heterogeneous and often based on small observational cohorts with variable follow-up, restricting definitive conclusions regarding long-term fertility implications. Additionally, many clinical studies rely on detection of viral RNA rather than replication-competent virus, which may overestimate the duration of infectious risk. Finally, evolving epidemiology and guidance mean that recommendations summarised here may require future revision as new evidence emerges. Despite these limitations, the available evidence provides a clinically relevant framework for counselling, risk stratification and fertility practice in men with current or previous ZIKV exposure.

## Conclusion

ZIKV ‘s ability to localise within the male reproductive tract and adversely impact semen quality represents an ongoing concern within the andrology and reproductive medicine communities. With steady epidemiological activity and potential for travel-related exposure, its diagnosis should be considered in those of reproductive age and at risk of exposure.

Key recommendations central to contemporary practice include identifying exposure risks within the clinical history, adherence to deferral intervals before conception or ART, and rigorous laboratory handling and closed-system cryostorage. Following these principles, most couples will be able to proceed safely and confidently with their fertility goals.

## Supplementary Information

Supplementary Material 1.

## Data Availability

Not applicable.

## Abbreviations

ART: Assisted reproductive techniques
BTB: Blood-testis barrier
CDC: Centres for Disease Control and Prevention
ECDC: European Centre for Disease Prevention and Control
PAHO: Pan American Health Organization
PCR / RT: PCR-Polymerase chain reaction / Reverse-transcription polymerase chain reaction
TORCH: Toxoplasmosis, Other (syphilis, varicella-zoster, parvovirus B19), Rubella, Cytomegalovirus, Herpes simplex
UKHSA: UK Health Security Agency
WHO: World Health Organization
ZIKV: Zika virus
