# Supplementary material for: Zika virus and male reproductive health: essential updates for andrologists and fertility specialists
Source: Basic Clin Androl. 2026 Mar 11;36:7. doi: 10.1186/s12610-026-00305-5 (PMC12977812; doi:10.1186/s12610-026-00305-5)
Supplement: Supplementary file 1 — Supplementary Material 1. [file 12610_2026_305_MOESM1_ESM.docx]

**Supplementary Appendix 1**

**Electronic Search Strategy and Public Health Sources**

**Database Search**

A structured literature search was conducted using PubMed as the primary biomedical database, covering publications from January 2015 to September 2025.

**Core Search Strategy**

The following search string represents the principal electronic search strategy applied in PubMed:

("Zika virus" OR ZIKV) AND

("male reproductive health" OR fertility OR andrology OR "assisted reproduction") AND

(semen OR testosterone OR spermatogenesis OR "sexual transmission")

Additional modifiers such as epidemiology and WHO guidance were applied where relevant to capture public health and clinical guidance literature. Search terms were adapted iteratively to reflect emerging themes identified during title, abstract, and full-text screening.

**Public Health and Guideline Sources**

To supplement peer-reviewed literature, epidemiological reports and clinical guidance were retrieved from the following recognised international and national health agencies:

- World Health Organization (WHO)
- Pan American Health Organization (PAHO)
- European Centre for Disease Prevention and Control (ECDC)
- UK Health Security Agency (UKHSA)
- Centers for Disease Control and Prevention (CDC)

These sources were consulted to ensure inclusion of contemporaneous surveillance data, reproductive health guidance, and recommendations relevant to fertility and andrology practice.

**Study Identification and Selection**

Reference lists of relevant publications were manually screened to identify additional sources. Primary research articles, review articles, epidemiological summaries, and official guidance documents were included where thematically relevant to ZIKV epidemiology, viral persistence in semen, testicular pathophysiology, and fertility-related clinical practice. Only English-language and accessible sources were retained. Evidence was synthesised narratively in accordance with the aims of the review.
